# A hybrid of B and T lymphoblastic cell line could potentially substitute dendritic cells to efficiently expand out Her-2/neu-specific cytotoxic T lymphocytes from advanced breast cancer patients in vitro

**DOI:** 10.1186/s13045-017-0429-8

**Published:** 2017-02-28

**Authors:** Sheng Chen, Feifei Gu, Kang Li, Kai Zhang, Yangyang Liu, Jinyan Liang, Wei Gao, Gang Wu, Li Liu

**Affiliations:** 1 Cancer Center, Union Hospital, Tongji Medical College, Huazhong University of Science and Technology, Wuhan, 430022 China; 2 Traumatology Department, Tongji Hospital, Tongji Medical College, Huazhong University of Science and Technology, Wuhan, 430022 China

**Keywords:** Hybrid of B and T lymphoblastic cell line, Adoptive cancer immunotherapy, Cytotoxic T lymphocytes, Breast cancer

## Abstract

Adoptive transfer of cytotoxic T lymphocytes (CTLs) holds promises to cure cancer. However, this treatment is hindered by lacking a robust way to specifically expand out CTLs. Here, we developed a hybrid of B lymphoblastic cell line and T lymphoblastic cell line (T2 cells) as a substitute of dendritic cells, together with irradiated autologous peripheral blood mononuclear cell (PBMC) as feeder cells and rhIL-2, to activate and expand Her-2/neu-specific CD8^+^ T cells from human epidermal growth factor receptor 2 (Her-2/neu) and human leukocyte antigen (HLA)-A2 double positive advanced breast cancer patients in vitro. These Her-2/neu-loaded T2 cells reproducibly activated and expanded out Her-2/neu-specific CD8^+^ T cells to 10^7^ in 8 weeks. Furthermore, these Her-2/neu-specific CD8^+^ T cells had good sensitivity of recognition and killing Her-2/neu-overexpressed breast cancer cell line SK.BR.3. This technique gives us another insight on how to rapidly obtain sufficient CTLs for adoptive cancer immunotherapy.

**Electronic supplementary material:**

The online version of this article (doi:10.1186/s13045-017-0429-8) contains supplementary material, which is available to authorized users.

To the editor

The current standard way to expand specific cytotoxic T lymphocytes (CTLs) replies obtaining sufficient dendritic cells (DCs) from patients (Additional file 1). This method has several defects such as invasive, time-consuming, expansive, and unstable according to patients’ physical conditions [1–5]. Our group found that the T2 cells, which are a cloned hybrid between the 721.174 (variant of the B lymphoblastic cell line LCL 721) and CEM^R^.3 (variant of T lymphoblastic cell line CEM-C7), potentially conform to the demands. The cells are TAP and MHC class II deficient, but they do express HLA-A2 and massive co-stimulatory molecules (Additional file 2: Figure S1). These characters make T2 cells potentially useful to study CD8^+^ T cell recognition of MHC class I antigens, meanwhile, convenient to exclude from MHC class II antigen intervention. Subsequently, HER-2/neu_(369–377)_ and HER-2/neu_(435–443)_ which scores more than 20 by SYFPEITHI prediction was selected to load to T2 cells to expand Her-2/neu-specific CD8^+^ T cells. HIV gag_(77–85)_, insulin B chain_(34–42),_ and HER-2/neu_(39–47)_ which scores minus 3 were performed as a control (see Additional file 1: Table S1). All peptides except low-affinity peptide HER-2/neu_(39–47)_ could stabilize HLA-A2 molecules on the cell membrane obviously (Additional file 3: Figure S2A).

After co-culture with CD8^+^ T cells from Her-2/neu and HLA-A2 double positive advanced breast cancer patients, HER-2/neu_(369–377)_- and HER-2/neu_(435–443)_-loaded T2 cells lead to a large secretion of IFN-γ, but not the three controls (Additional file 3: Figure S2B).

For the expansion, starting from 1 × 10^5^ total CD8^+^ T cells that were less than 0.05% Her-2/neu-specific, nearly 10^7^ HER-2/neu_(369–377)_ or HER-2/neu_(435–443)_-specific CD8^+^ T cells were expanded out in 8 weeks with the purity of around 85% (Fig. 1a, b). The expanded specific CD8^+^ T cells got massive co-stimulatory molecules and activation makers (Fig. 1c). The three controls got negative results. That was expected due to extremely low frequency of naturally occurring HIV gag_(77–85)_ and insulin B chain_(34–42)_-specific naive CD8^+^ T cells as indicated in Additional file 3: Figure S2B and low affinity HER-2/neu_(39–47)_ peptides as showed in Additional file 1: Table S1. We found these expanded HER-2/neu-specific CD8^+^ T cells were mainly effector and effector memory cells (Additional file 4: Figure S3).

**Fig. 1:**
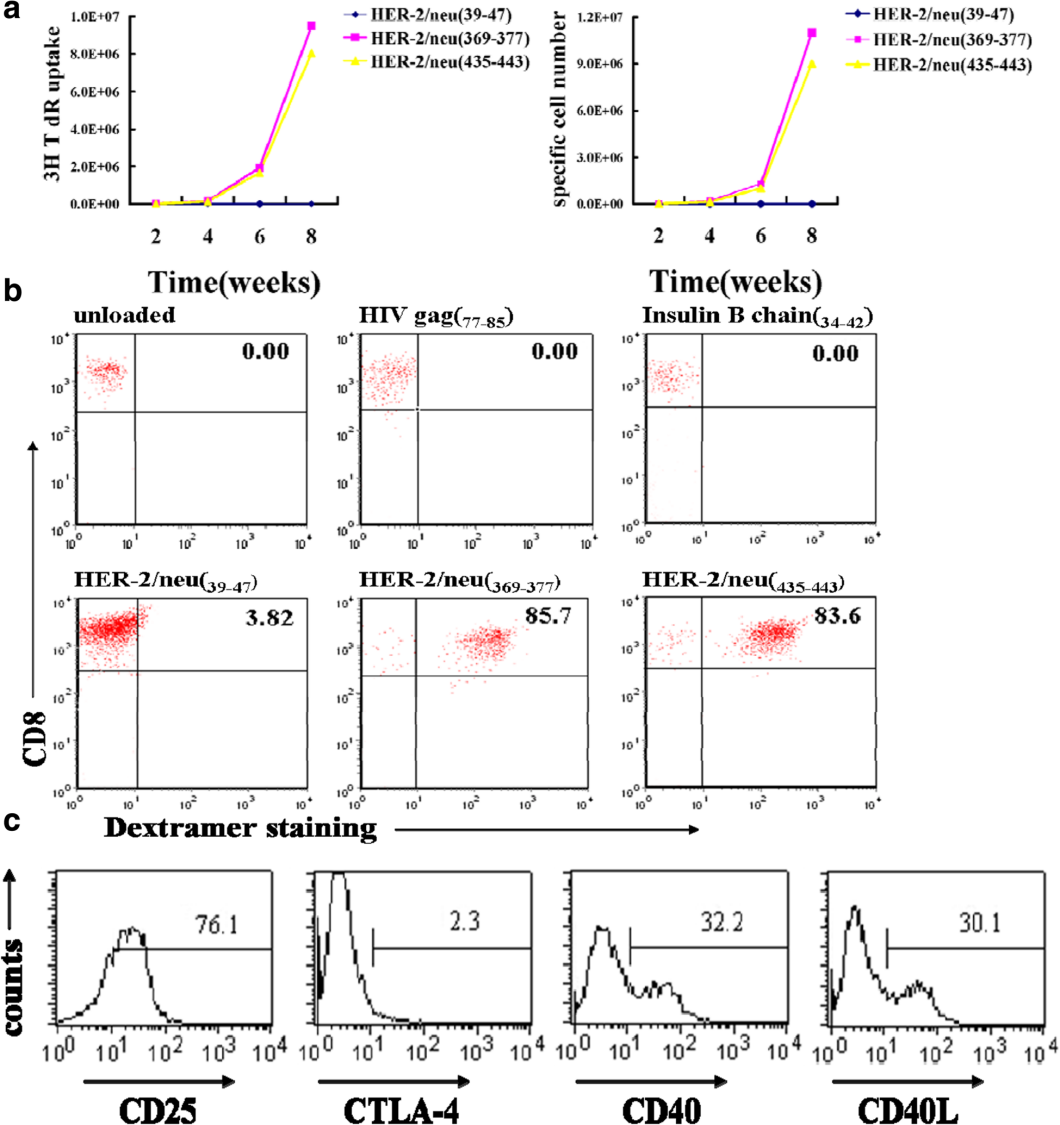
Her-2/neu-specific CD8^+^ T cells are efficiently expanded out by Her-2/neu-loaded T2 cells from advanced breast cancer patients. **a** In vitro Her-2/neu-specific CD8^+^ T cells were expanded out by Her-2/neu-loaded T2 cells in addition with rhIL-2 and irradiated autologous PBMC (peptides, HER-2/neu_(39–47)_, HER-2/neu_(369–377)_, and HER-2/neu_(435–443)_, respectively). *Left*, proliferation assessed as uptake of [^3^H] thymidine; *right*, total cell number counting of specific CD8^+^ T cells after expansion. Nearly 1.0E + 07 Her-2/neu-specific CTLs were expanded out in 8 weeks. **b** Her-2/neu-specific CD8^+^ T cells were verified by Her-2/neu-conjugated HLA-A*0201 Dextramer staining by fluoresence. Her-2/neu_(369–377)_- and Her-2/neu_(435–443)_-loaded T2 cells expanded out specific CD8^+^ T cells with the purity of around 85%. **c** The expanded Her-2/neu specific CD8^+^ T cells expressed massive activation makers CD25, CD40, and CD40L. The results are all from one representative of three independent experiments

The expanded Her-2/neu-specific CTL populations mediated dose-dependent lysis to HLA-A2-positive and Her-2/neu-overexpressed breast cancer cell line SK.BR.3, but not to the three control targets (Fig. 2a). The lysis attributed to over 50% perforin producing CTLs and more than 80% granzyme B producing CTLs (Fig. 2b). The cytotoxic activity against SK.BR.3 could significantly be eliminated by W6/32, but not by IgG2 (shown in Fig. 2c).

**Fig. 2:**
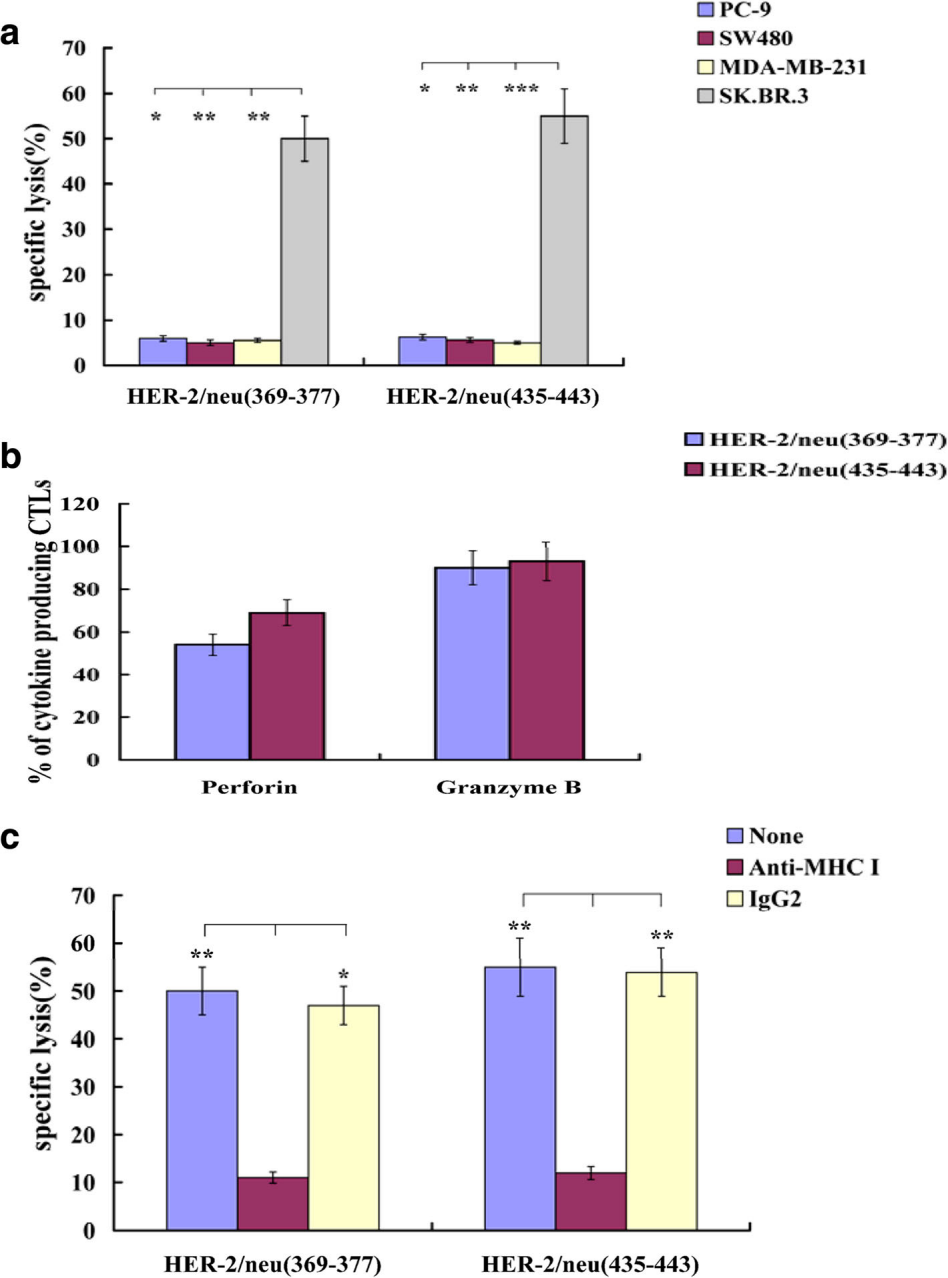
The expanded Her2/neu-specific CTLs well recognize and kill the related targets. **a** The expanded effector cells (HER-2/neu_(369–377)_-or HER-2/neu_(435–443)_-specific CD8^+^ T cells) could only lyse HLA-A2-positive and Her-2/neu-overexpressed breast cancer cell line SK.BR.3, but not the controls. **b** Perforin or granzyme B secretion by expanded Her2/neu-specific effector cells was detected after co-culture with SK.BR.3. **c** Anti-HLA class I antibody blocking inhibited the cytotoxic activity of the expanded Her-2/neu-specific CD8^+^ T cells against SK.BR.3. Mouse IgG2 antibody was added as a control. *P < 0.05, **P < 0.01, ***P < 0.001 (ANOVA)

A major finding of this study was that the Her-2/neu-loaded T2 cells could expand out nearly 10^7^ Her-2/neu-specific CTLs in 8 weeks. The expanding efficiency is equivalent to DCs previously reported by Marzocchetti et al. [6–8]. And because the expansion was started from CD8^+^ T cells which were only from 5 ml blood, with an initial frequency of Her-2/neu-specific CD8^+^ T cells at 0.05%, it would be possible to amplify the expanding quantity if we isolate the Her-2/neu-specific CD8^+^ T cells firstly from more blood, 50 ml or more for example.

We found the expanded HER-2/neu-specific CTLs could recognize endogenous antigen on allogeneic breast cancer cell line SK.BR.3. This is critically important because previously, many expanding techniques lead to CTLs that could only kill targets pulsed with related peptides but not targets that endogenously processed the antigen of interest [9]. Yee et al. [10, 11] found low affinity of induced CTLs leads to failure of recognition of endogenous antigen.

Thus, T2 cells have shown promise as a convenient tool to rapidly expand out Her-2/neu-specific CTLs in vitro. But so far, we are not able to transfer the expanded Her-2/neu-specific CTLs to breast cancer patients directly as they are mixed with T2 cells, and for the same reason, we cannot compare its anti-tumor effect with trastuzumab in breast cancer patients either. This technique might accelerate to study expanding CTLs in vitro and promote the development of safe and effective adoptive cancer immunotherapy in the future.

## Additional files

Additional file 1: Materials and methods. (DOC 199 kb)
Additional file 2: Figure S1. T2 cells express more HLA-A2 and equivalent co-stimulatory molecules compared with DC cells. (TIF 173 kb)
Additional file 3: Figure S2. Her-2/neu-loaded T2 cells can activate related CD8^+^ T cells equally as DCs. a T2 cells stabilized the MHC I molecules on the cell membrane after loading relative restricted peptides. The mean fluorescence intensity of HLA-A2 was detected by FACS staining before or after peptide loading. b Her-2/neu-loaded T2 cells could activate CD8^+^ T cells as effectively as DC cells. IFN-γ secretion of CD8^+^ T cells after activation was detected by ELISA. **P* < 0.05, ***P* < 0.01 (Student’s *t* test). (TIF 14741 kb)
Additional file 4: Figure S3. The expanded Her-2/neu-specific CD8^+^ T cells are mainly effector and effector memory cells. CCR7 and CD45RA expression on expanded CD8^+^ T cells was detected by FACS. The expanded Her-2/neu specific CTLs were partially CCR7^−^CD45RA^+^ (effector), partially CCR7^−^CD45RA^−^ (effector memory), and rarely (about 1.62%) CCR7^+^CD45RA^+^ (naive). This representative data are from the expanded Her-2/neu_(369–377)_-specific CD8^+^ T cells. (TIF 11635 kb)

## Abbreviations

CCR7: C-C chemokine receptor type 7
CD45: Cluster of differentiation 45
CTLs: Cytotoxic T lymphocytes
DCs: Dendritic cells
Her-2/neu: Human epidermal growth factor receptor 2
HIV: Human immunodeficiency virus
HLA: Human leukocyte antigen
IL-2: Interleukin-2
MHC: Major histocompatibility complex
PBMC: Peripheral blood mononuclear cell
TCR: T cell receptor
