# Supplementary material for: A hybrid of B and T lymphoblastic cell line could potentially substitute dendritic cells to efficiently expand out Her-2/neu-specific cytotoxic T lymphocytes from advanced breast cancer patients in vitro
Source: J Hematol Oncol. 2017 Feb 28;10:63. doi: 10.1186/s13045-017-0429-8 (PMC5331710; doi:10.1186/s13045-017-0429-8)
Supplement: Additional file 1: — Materials and methods. (DOC 199 kb) [file 13045_2017_429_MOESM1_ESM.doc]

**Materials and methods**

Cell lines, antibodies and cytokines

T2 cells purchased from ATCC were cultured in RPMI-1640 containing 10% FCS. Anti-CD3-FITC(Becton Dickinson Biosciences), anti-CD8α-PE(RPA-T8)(Biolegend), anti-CCR7-PE-Cy7(BD Biosciences), anti-CD45RA-Alexa405(BD Biosciences), anti-perforin or granzyme B-FITC(BD Biosciences), anti-HLA-A2 (BB7.2)(Abcam), anti-CD3(OKT3)(Biolegend), anti-CD28(15E8)(Millipore), and Mouse IgG2a isotype control(HOPC-1)(Southern Biotech) were all purchased from related commercial company. Biological properties of all antibodies were mouse anti-human. Human GM-CSF, recombinant human IL-2, IL-7, and IL-15 were purchased from PeproTech.

Peptides and SYFPEITHI prediction

HER-2/neu_(369-377)_[KIFGSLAFL],HER-2/neu_(435-443)_[ILHNGAYSL],HER-2/neu_(39-47)_[PETHLDMLR]from human epidermal growth factor receptor 2, HIV gag_(77-85)_[SLFNTIATL]from human immunodeficiency virus Gag protein, and Insulin B chain_(34-42)_[HLVEALYLV] were chemically synthesized by Invitrogen and verified by mass spectrometry.

The SYFPEITHI prediction values of all possible nonamers of the sequence of HER-2/neu were added together. Scores for predicted epitopes specifically for HLA-A*0201 were given in (Tab. 1). The SYFPEITHI prediction scores of these epitopes were usually higher than 20, and HIV gag_(77-85)_ [SLFNTIATL], Insulin B chain_(34-42)_ [HLVEALYLV] and the peptide HER-2/neu_(39-47)_ [PETHLDMLR]which scores minus 3 were performed as a control.

**Tab. 1** HLA-A*0201 Restricted HER-2/neu Epitope Prediction Results


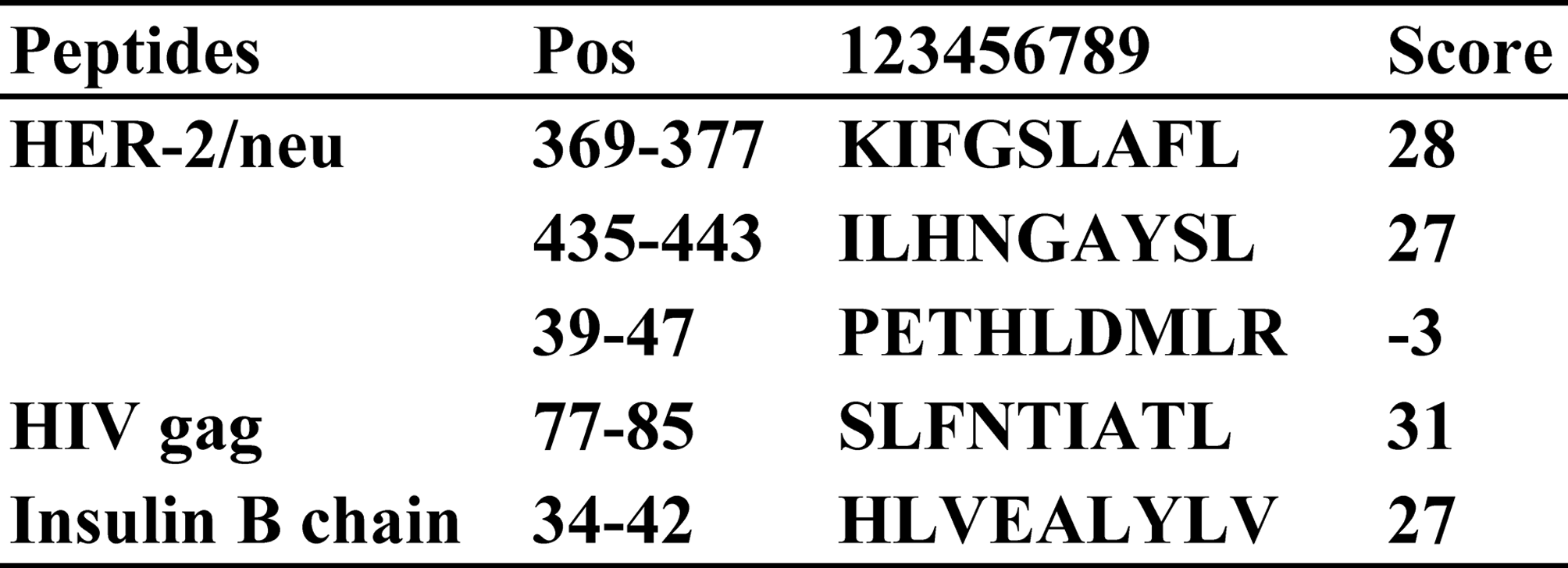


Patients and CD8^+^ T cells obtainment

10 advanced breast cancer patients which were Her-2/neu and HLA-A*0201 double positive were selected to complete the study with their written consent. Her-2/neu was deemed as positive if its expression in tumor tissue was 3+ by immunohistochemistry or positive by fluorescence in situ hybridization, FISH. HLA-A*0201 was deemed as positive if PBMC was stained as positive by anti-HLA-A2 (BB7.2) by FACS. For contrasting, these patients were all free from Type 1 diabetes and HIV infection according to related clinical diagnosis.5ml venous blood was collected via drawing of vena mediana basilica from the patients. PBMC was isolated from the blood by means of density gradient centrifugation in LSM 1077 separation medium. CD8^+^ T cells isolation from PBMC was performed with Dynal Untouched Human CD8^+^ T cell kit according to the instructions of manufacturer. Both PBMC and CD8^+^ T cells were cultured in RPMI-1640 with 10% AB human serum.

CD8^+^ T cells activation, expansion and maintenance

For the activation, (10^5^ cells/well) CD8^+^ T cells were then co-cultured with 10^4^ peptide-pulsed T2 cells in 96-well round-bottom plates in 200μl/well medium. Two days later, the supernatant was detected for IFN-γ secretion by ELISA. For the expansion, 10^4^ peptide-pulsed T2 cells were radiated with 20Gy X ray firstly, then co-cultured with (10^5^ cells/well) CD8^+^ T cells. Additionally, 10^4^ irradiated autologous PBMC (20Gy by X ray as well) as feeder cells and rhIL-2 were supplemented to the culture system. The rhIL-2 was in final work concentration of 20ng/ml. On day 14 and biweekly thereafter, CD8^+^ T cells were collected, counted and re-plated at 10^5^ T cells per well, together with 10^4^ irradiated peptide-pulsed T2 cells, 10^4^ irradiated autologous PBMC and the above rhIL-2. Finally, the expanded CD8^+^ T cells were maintained in the above medium, with biweekly stimulation with α-CD3 antibodies (OKT3) and rhIL-2.

[^3^H]thymidine uptake

[^3^H]thymidine was added to the proliferated CD8^+^ T cells at a dose of 1 μCi per well. And [^3^H]thymidine uptake was performed after 12-16 hours of co-culture according to the manufacturer’s protocols.

Flow cytometry and FACS sorting

Cells were stained with antibodies at 4°C, and analysed on FACS Calibur flow cytometer(BD Biosciences). Cell sorting was performed on a MoFlo cell sorter (Cytomation, Fort Collins).

Dextramer production and staining

FITC labeled, and HER-2/neu conjugated HLA-A*0201 Dextramer (IMMUDEX) was used to stain expanded HER-2/neu specific CD8^+^ T cells. Cells were stained at room temperature, and measured by using a FACS Calibur flow cytometer.

^51^Cr-release assay

To detect the cytotoxic activity of the T2-expanded CTLs against target cells, ^51^Cr-labeled (500_Ci) target cells were co-cocultured with effector cells at effector target ratios at 25:1 at 37°C for 4 hours. Radioactivity was counted from an aliquot of supernatant. CTL cytotoxicity activity was calculated as the percentage of specific ^51^Cr release with the following equation: percent specific killing=(sample release-spontaneous release)÷(maximal release-spontaneous release)×100.

Statistical analysis

Statistical analysis of two samples such as cytokine production and CD8^+^ T cell proliferation was evaluated by a Student’s t-test with 2 sample equal variance with a 2-tailed distribution. Multiple samples tests such as the ^51^Cr-release assay and subsequent inhibition of cytotoxicity of CTLs were evaluated by the one-way analysis of variance (ANOVA) followed by the Tukey post test. Data are showed as mean±SD. Statistical significance was set at P<0.05.
