# Supplementary figures and images for: A hybrid of B and T lymphoblastic cell line could potentially substitute dendritic cells to efficiently expand out Her-2/neu-specific cytotoxic T lymphocytes from advanced breast cancer patients in vitro
Source: J Hematol Oncol. 2017 Feb 28;10:63. doi: 10.1186/s13045-017-0429-8 (PMC5331710; doi:10.1186/s13045-017-0429-8)

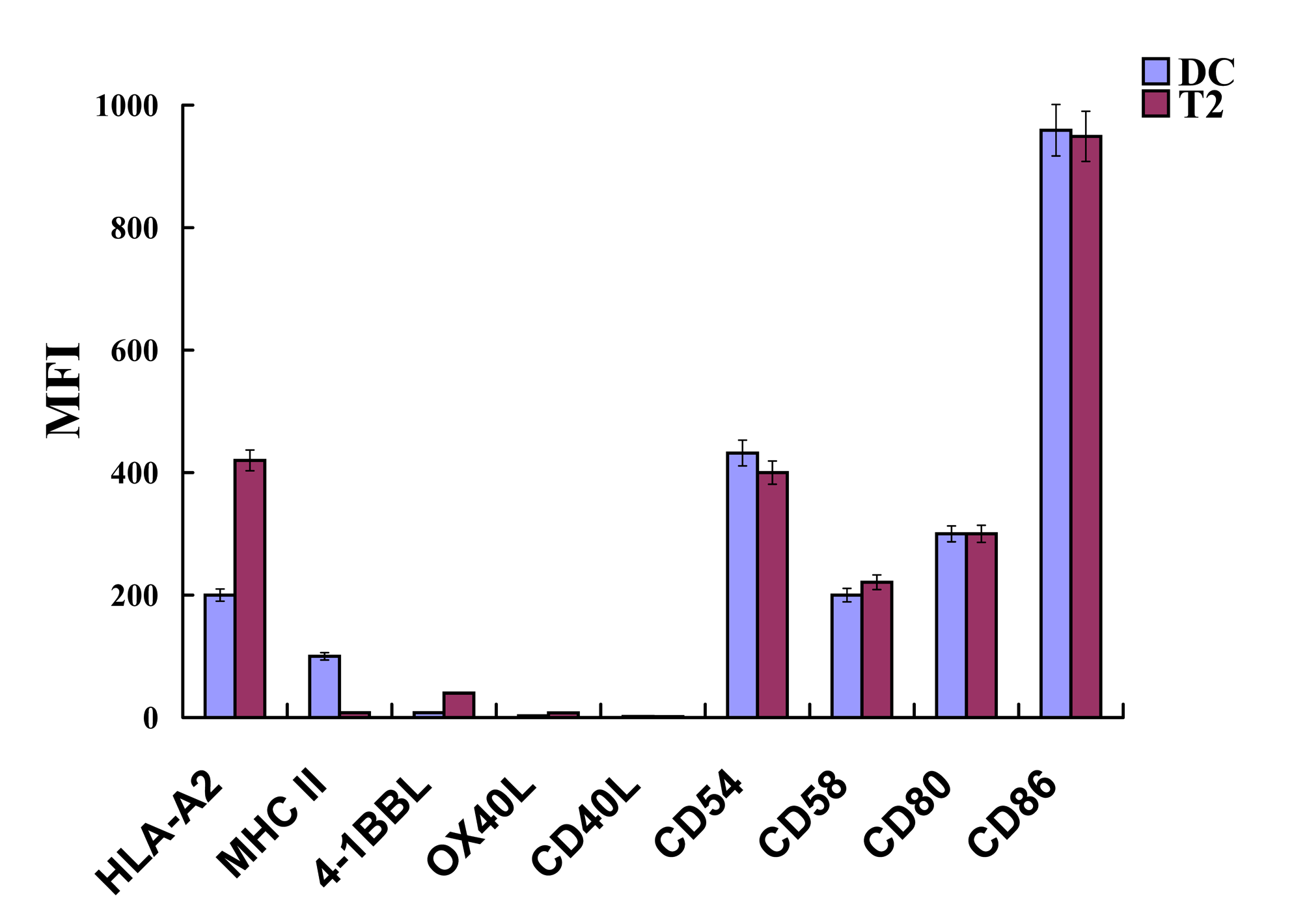

Supplement: Additional file 2: Figure S1. — T2 cells express more HLA-A2 and equivalent co-stimulatory molecules compared with DC cells. (TIF 173 kb) [file 13045_2017_429_MOESM2_ESM.tif]

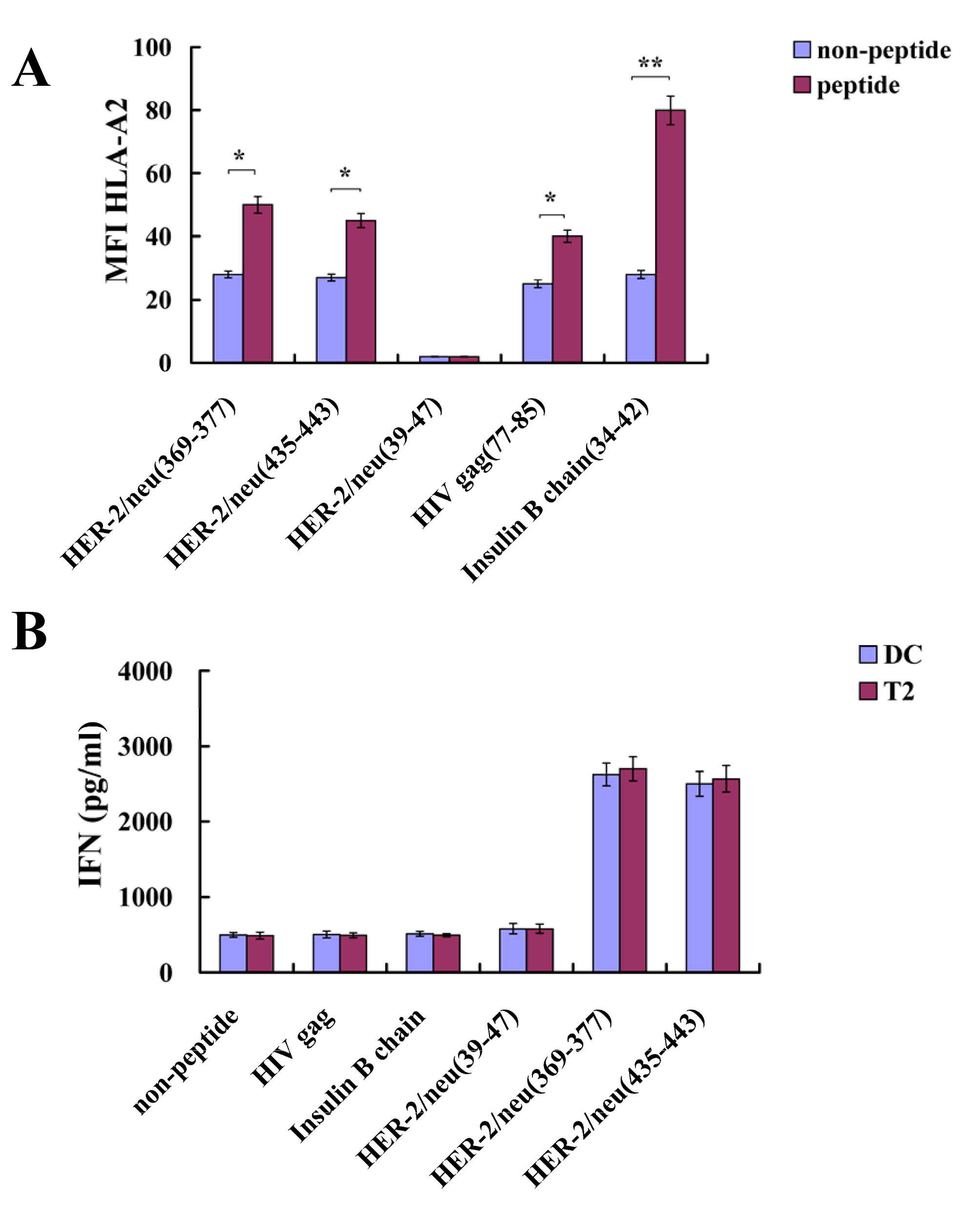

Supplement: Additional file 3: Figure S2. — Her-2/neu-loaded T2 cells can activate related CD8+ T cells equally as DCs. a T2 cells stabilized the MHC I molecules on the cell membrane after loading relative restricted peptides. The mean fluorescence intensity of HLA-A2 was detected by FACS staining before or after peptide loading. b Her-2/neu-loaded T2 cells could activate CD8+ T cells as effectively as DC cells. IFN-γ secretion of CD8+ T cells after activation was detected by ELISA. *P < 0.05, **P < 0.01 (Student’s t test). (TIF 14741 kb) [file 13045_2017_429_MOESM3_ESM.tif]

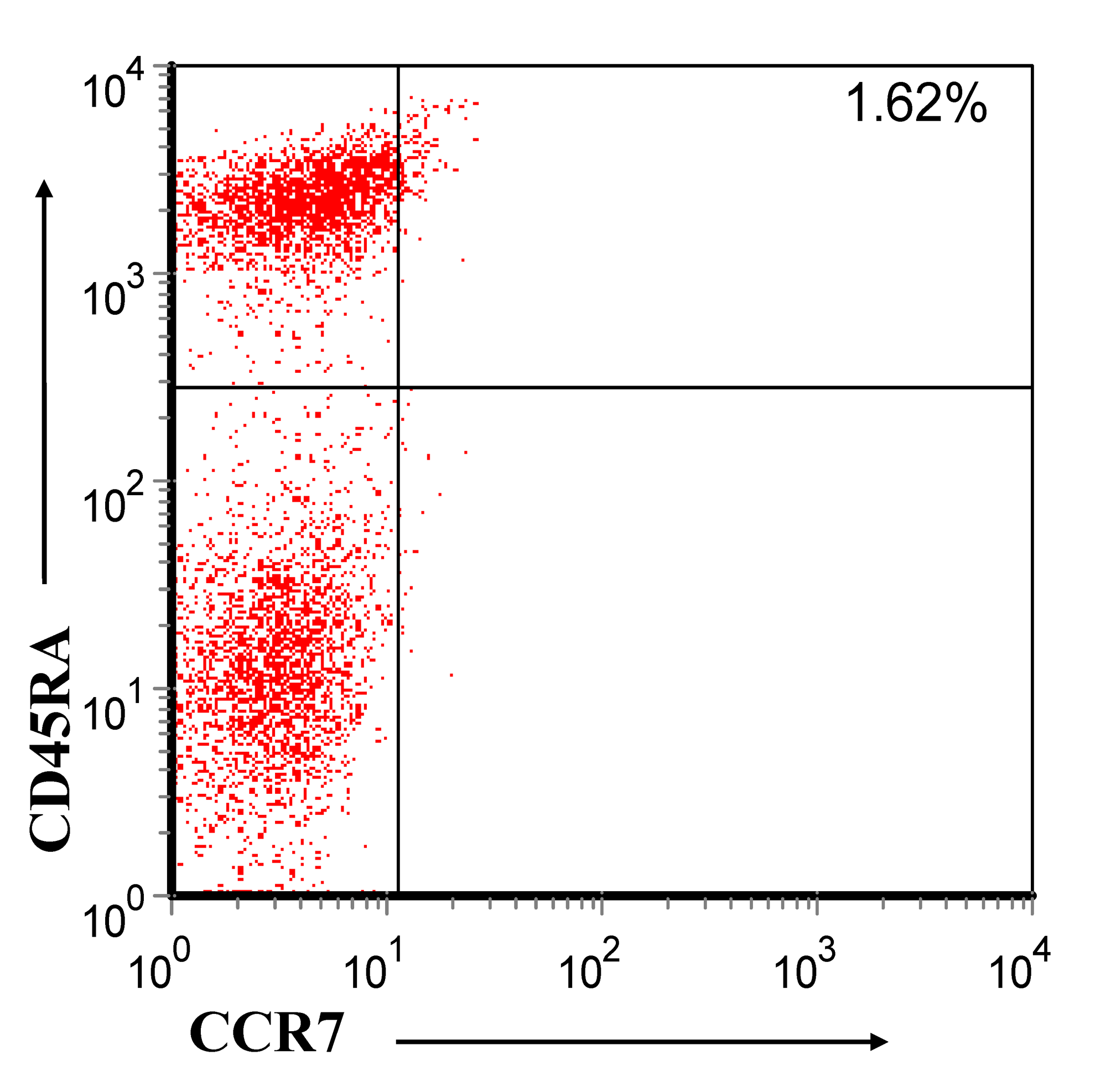

Supplement: Additional file 4: Figure S3. — The expanded Her-2/neu-specific CD8+ T cells are mainly effector and effector memory cells. CCR7 and CD45RA expression on expanded CD8+ T cells was detected by FACS. The expanded Her-2/neu specific CTLs were partially CCR7−CD45RA+ (effector), partially CCR7−CD45RA− (effector memory), and rarely (about 1.62%) CCR7+CD45RA+ (naive). This representative data are from the expanded Her-2/neu(369–377)-specific CD8+ T cells. (TIF 11635 kb) [file 13045_2017_429_MOESM4_ESM.tif]
